# Thermodynamics-based Cognitive Demodulation for 'THz Torch' Wireless Communications Links

**DOI:** 10.1038/s41598-020-62234-1

**Published:** 2020-04-10

**Authors:** Hang Ren, Stepan Lucyszyn

**Affiliations:** Department of Electrical and Electronic Engineering, Imperial College London, London, SW7 2AZ UK

**Keywords:** Electrical and electronic engineering, Lasers, LEDs and light sources

## Abstract

The low-cost ‘THz Torch’ technology, which exploits the thermal infrared spectrum (*ca*. 10 to 100 THz), was recently introduced to provide secure low data rate communications links across short distances. In this paper, a thermodynamics-based approach is proposed for greatly enhancing the sensitivity of detection with non-stationary thermal radiation, generated by thermal emitters that have been modulated well beyond their thermal time constants. Here, cognitive demodulation is employed and, unlike all previous demonstrators, allows truly asynchronous operation by dynamically predicting the thermal transients for the next bit to be received. The result is a five-fold increase in the reported operational figure of merit (Range  ×  Bit Rate) for ‘THz Torch’ wireless communications links. A single-channel (2 m  ×  125 bps) prototype and an 8-channel frequency-division multiplexed (0.5 m  ×  1,000 bps) prototype are demonstrated as proof-of-principle exemplars for the enhanced method of demodulation. Measurements show superior bit error rate performance with an increase in range and bit rate, when compared with conventional threshold detection. This work represents a paradigm shift in thermal-based modulation-demodulation of digital data, and offers a practical solution for the implementation of future ubiquitous secure ‘THz Torch’ wireless communications links; as well as other applications.

## Introduction

Wireless links represent the fastest growing area within the digital communications industry. The history of transmitting data wirelessly can be traced back to ancient China, using smoke signaling for long-distance communication. Other early examples of wireless communications include optical telegraphy (Heliography) from the 19th century and photophone in the early 20th century^1^. Today, wireless communications systems play a crucial role in every aspects of human life.

In general, wireless communications systems can be found in most parts of the electromagnetic (EM) spectrum; at radio frequencies that extend upwards into the far-infrared (i.e., covering the (sub-)microwave^2^, millimeter-wave^3^ and (sub)-terahertz bands^4,5^) and at optical wavelengths that extend downwards (i.e., across the visible light spectrum^6^ into the near-infrared^7^). However, little research and development has been reported on thermal infrared (i.e., around the near-infrared, having atmospheric transmission windows from 20–40 THz and 60–100 THz) wireless communications links. Until recently, the thermal infrared spectrum has been generally confined to applications that include motion sensing, target acquisition^8^ and thermography^9^ (e.g., digital thermometers and thermal cameras); having relatively low-cost components that work with incoherent radiation, when compared to extremely expensive components used in coherent systems^10^ operating at radio frequencies and optical wavelengths.

In 2011, the first thermal infrared wireless communications system^11^ was reported, which exploited the use of extremely low-cost thermal emitters (miniature incandescent light bulb arrays) and pyroelectric infrared (PIR) motion sensors; collectively referred to as the ‘THz Torch’ technology within the physical layer. Over the past nine years, various aspects have been investigated, from systems level architectures^12^ down to device level enabling technologies^13–15^, while maintaining the underlying ethos of employing inherently low-cost components − making the technology affordable for future ubiquitous low data rate wireless links that can be used for secure applications (e.g., private key fobs, covert remote controls, secure device-to-device data links, etc.). Proof-of-principle single^11,16^ and multi-channel^12,17^ demonstrations have been previously reported.

The first reported demonstrator^11^ employed a direct electronic modulation scheme, having the advantages of simplicity, reliability and low cost, but the maximum bit rate for sending arbitrary data is of the order of 10 bps. With subsequent prototypes^12,15–17^, the emitters generated a constant level of thermal radiation power (i.e., time-invariant) and employed mechanical optical choppers as an indirect modulation mechanism. This solution mitigates against thermal time constant constraints, offering the advantage of faster data rates. Maximum bit rates for sending arbitrary data are of the order of 640 bps using mechanical optical choppers^17^, limited by the moment of inertia and angular momentum; faster speeds are possible with mechanical optical shutters. However, all mechanical solutions are expensive and have limited working lifetimes of operation (short-lifetimes with shutters and medium-lifetimes with choppers). To date, the reported operational figure of merit (Range  ×  Bit Rate) for ‘THz Torch’ wireless communications links have been limited by either thermal mass (direct electronic modulation) or physical mass (indirect mechanical modulation). With the former, solid-state solutions may be needed; with the latter, one possible solution could be to employ microelectromechanical systems (MEMS) technology.

If conventional threshold detection (based simply on detecting a change in output voltage from a PIR sensor), is replaced by one that dynamically predicts the thermal transients for the two possible states of the next bit to be received (referred to here as ‘cognitive demodulation’) then it is possible to dramatically increase the operational figure of merit. In this paper, the basic principles behind the ‘THz Torch’ technology is redeveloped from a stationary radiation perspective (with operation constrained by the inherent thermal time constants associated with the thermal source and/or sensor) to one that truly exploits natural thermodynamic behaviour. The underlying theory is proved experimentally with both single-channel and multi-channel prototypes, demonstrating significantly reduced bit error rate (BER) with increased transmission range and bit rate.

## Results

**Figure 1:**
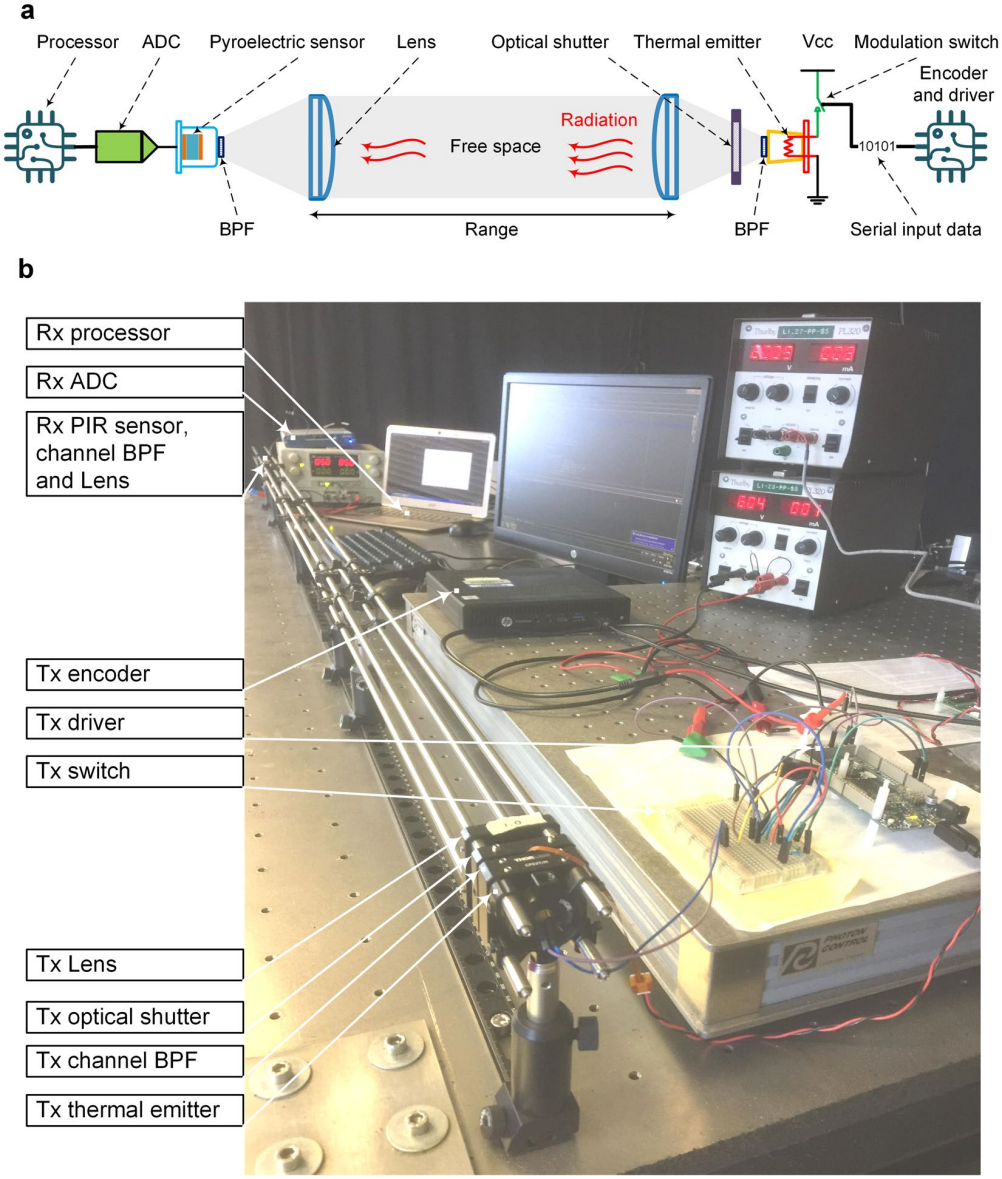
Typical ‘THz Torch’ wireless channel. (**a**) Architecture. (**b**) Experimental setup with a range of 2 m.

The typical architecture for a ‘THz Torch’ wireless channel is illustrated in Fig. 1a, consisting of a transmitter (Tx) and receiver (Rx); the corresponding experimental setup for a 2 m link is shown in Fig. 1b. The thermal emitter is modulated using the simple ON-OFF keying (OOK) scheme by the input serial digital bit steam. The thermal radiation generated is spectrally filtered using an optical band pass filter (BPF), which defines the frequency range of the incoherent noise carrier for a specific channel (listed in Supplementary Table S1). To minimize spreading loss, the thermal emitter has a conical horn; a plano-convex optical lens is added to collimate the diverging beam of thermal radiation. After propagating through the free-space channel, the collimated thermal radiation is focused by an identical lens, through a matched BPF, onto a PIR sensor. The analogue voltage signal from the sensor is fed into an analogue-to-digital converter (ADC) and processor, from which the transmitted data is recovered. A mechanical optical shutter is included to generate a thermal radiation step function, needed for initial channel calibration.

### OOK modulated thermal infrared emitter

Conventional digital systems rely on linear memoryless modulation. One exception is the Miller code; this is a simple baseband line code (mapping bits to a real-valued analogue waveform) employing memory and nonlinear modulation^18^. With the Miller code, the waveform corresponding to the current bit is defined by two consecutive bits (the current bit and its previous bit). In contrast, with our system, we have a complex memory and nonlinear modulation (we simplistically refer to this as OOK), in which the transmit waveform is defined by the current bit and all previous bits within the same frame. Our uniquely thermal-based transmit physical layer naturally lends itself to our line code solution.

The ‘THz Torch’ transmitter employs a thermal emitter and a regulated power supply; connected via a binary switch, with ON/OFF state controlled by 100% duty cycle pulses corresponding to the input serial digital bit steam. The non-return-to-zero (NRZ) bit rate corresponds to the reciprocal of the pulse width duration. With OOK modulation, when transmitting a binary one (ON state), the power supply is connected to this crude thermal emitter, thereby heating it up during this clock period. When transmitting a binary zero (OFF state), the power supply is disconnected from the thermal emitter, thereby cooling it down naturally during this clock period.

**Figure 2:**
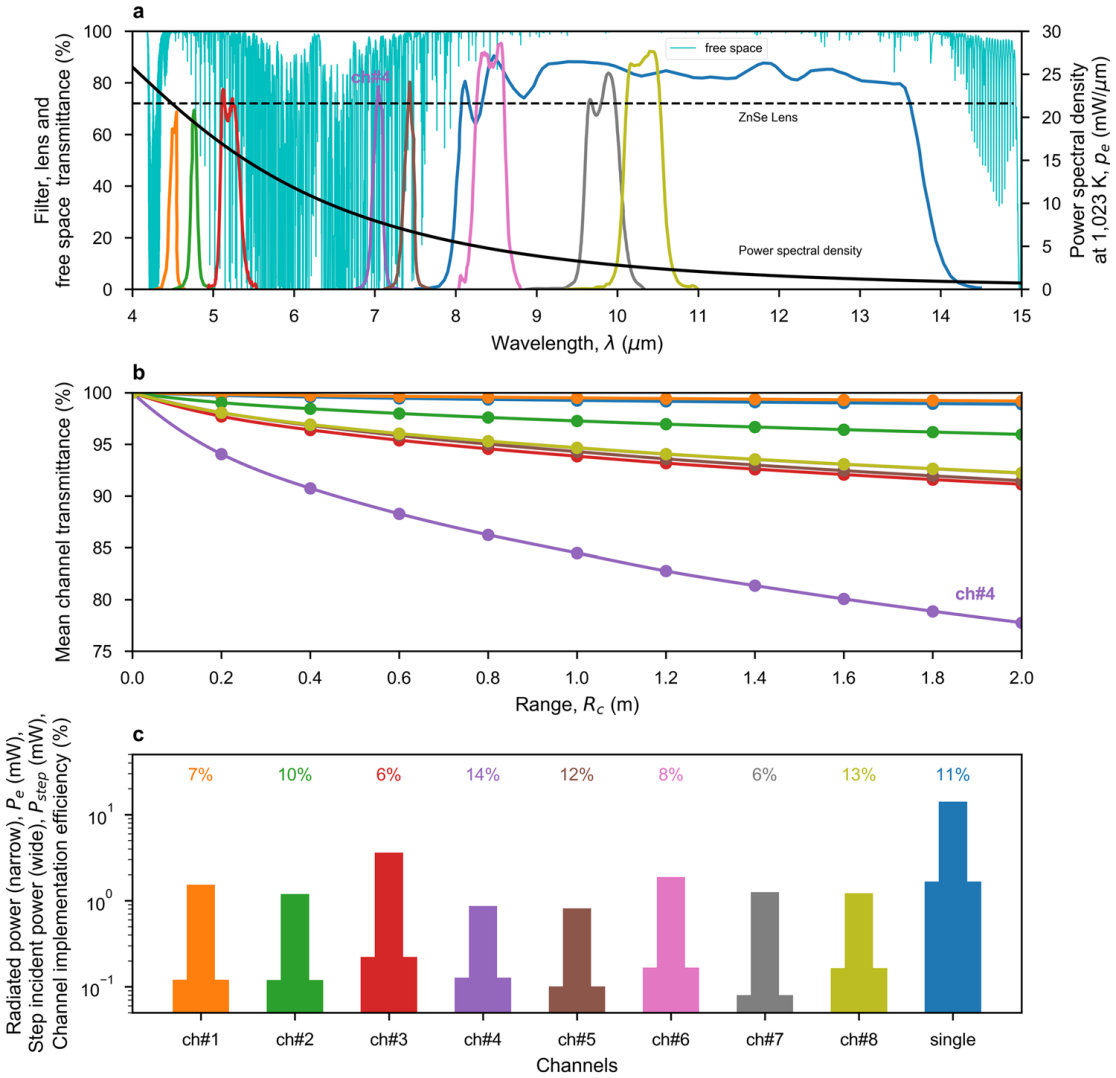
Channel characterization. (**a**) Theoretical thermal emitter power spectral density, measured channel band-pass filter and lens transmittance responses, and simulated free-space channel transmittance across a 2 m link under pristine conditions. (**b**) Predicted mean channel transmittance against range, using Eq. (6). (**c**) Theoretical radiated power within the channel filter passband, using Eq. (3), for a steady-state emitter temperature of 1,023 K; step incident power, using Eq. (15) with *L*_*c*_ = 1; and the resulting channel implementation efficiency.

The spectral radiance generated by an ideal blackbody emitter can be expressed by Planck’s law:

1 $$I(\lambda ,T)=\frac{2h{c}^{2}}{{\lambda }^{5}}\frac{1}{{e}^{hc/(\lambda {k}_{B}T)}-1}$$

where *λ* is the wavelength in free space; *h* is the Planck constant; *c* is the speed of light in free space; *T* is emitter temperature; and *k*_*B*_ is the Boltzmann constant. Unlike an isotopic source that radiates over a perfect sphere, our thermal emitter has a heated film (with area *A*_*e*_) that is better modelled by radiation over a half-sphere. This radiation is assumed to be efficiently confined by a reflecting conical horn, giving a 1/*e*^2^ angular beamwidth of only ±20° at 10 cm. Moreover, unlike a blackbody radiator, our emitter has an emissivity *ε*. As a result, the theoretical thermal emitter power spectral density *p*_*e*_ can be given as:

2 $${p}_{e}(\lambda ,T)=\varepsilon {A}_{e}I(\lambda ,T){\int }_{0}^{2\pi }cos(\theta )d\Omega ={\int }_{0}^{2\pi }d\varphi {\int }_{0}^{\frac{\pi }{2}}cos(\theta )sin(\theta )d\theta =\pi \varepsilon {A}_{e}I(\lambda ,T)$$

where *Ω* is the solid angle; *θ* and *φ* are the zenith and azimuth angles in spherical coordinates, satisfying *dΩ* = *sin*(*θ*)*dθdφ*. The *cos*(*θ*) term is due to the Lambertian nature of the thermal emitter. By integrating Eq. (2), over the wavelength of the defined channel, the theoretical thermal emitted radiated power of the noise carrier is given by:

3 $${P}_{e}(T)={\int }_{{\lambda }_{1}}^{{\lambda }_{2}}{p}_{e}(\lambda ,T)d\lambda $$

where, the lower and upper cut-off wavelengths for the channel are *λ*_1_ and *λ*_2_, respectively, extracted from Fig. 2a and given in Supplementary Table S1. The radiated power calculated using Eq. (3) is shown in Fig. 2c, for each channel.

With direct electronic modulation of the thermal emitter, its transient temperature *T*(*t*) varies between the extreme limits of the ambient temperature *T*_0_ and steady-state temperature *T*_*s*_. Obeying the principle of conservation of energy, while ignoring thermal convection, the following analytical expression has been formulated:

4 $${W}_{e}(t)={C}_{e}\frac{dT(t)}{dt}+{k}_{e}[T(t)-{T}_{0}]+\sigma \varepsilon {A}_{e}{T}^{4}(t)$$

where, *W*_*e*_(*t*) is the thermal emitter’s input power, being time-dependent with direct electronic modulation; *C*_*e*_ is the heat capacity of the thermal emitter; *k*_*e*_ is the thermal conductivity; *σ* is the Stefan-Boltzmann constant. Equation (4) has three terms that describe a dynamic process in which: input power contributes to a change in emitter temperature; thermal conduction (between the emitter and the environment); and black-body radiation. Using Eq. (4), the thermal conductivity *k*_*e*_ can be determined by substituting *W*_*e*_(*t*) with the manufacturer’s rated emitter DC power *W*_*R*_ and *T*(*t*) for *T*_*s*_; with this steady-state scenario, the temperature gradient goes to zero. A grid search method is then exploited to find a value of *C*_*e*_ that gives a predicted thermal time constant that equals the measured value given by the manufacturer.

Equation (4)  does not have a closed-form solution for the transient emitter temperature *T*(*t*) and so requires numerical methods, which could be problematic for implementing fast real-time demodulation. The power series method can be used to seek a solution to differential Eq. (4). After a lengthy derivation, the third-order power series presented in Eq. (5) represents a good approximation over a short duration ( ~ 12 ms in this case), which is computationally fast to implement.

5 $$\begin{array}{lll}T(t) & = & T(0)+{a}_{1}t+{a}_{2}{t}^{2}+{a}_{3}{t}^{3}+O({t}^{4})\\ {a}_{1} & = & \frac{{W}_{e}+{k}_{e}[{T}_{0}-T(0)]-\sigma \varepsilon {A}_{e}{T}^{4}(0)}{{C}_{e}}\\ {a}_{2} & = & -\frac{{a}_{1}[{k}_{e}+4\sigma \varepsilon {A}_{e}{T}^{3}(0)]}{2{C}_{e}}\\ {a}_{3} & = & -\frac{{a}_{2}{k}_{e}+4{a}_{2}\sigma \varepsilon {A}_{e}{T}^{3}(0)+6{a}_{1}^{2}\sigma \varepsilon {A}_{e}{T}^{2}(0)}{3{C}_{e}}\end{array}$$

**Figure 3:**
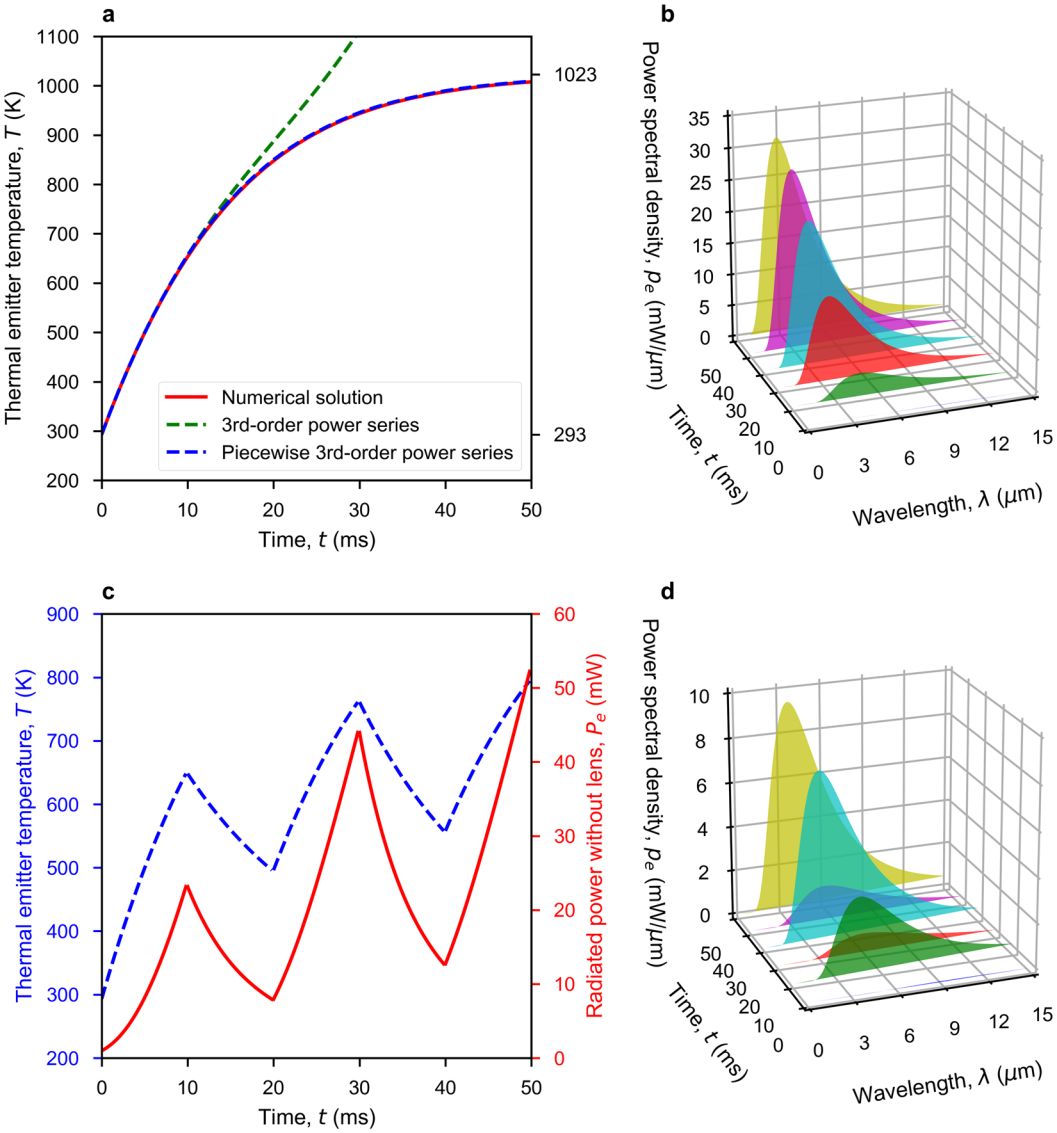
Calculated ideal thermal emitter (INTX 17-0900) responses. (**a**) Transient turn-ON behaviour of emitter temperature, with 898 mW of emitter DC power, from OFF-state at 20 ^o^C ambient room temperature. (**b**) Time-variant power spectral density of thermal radiation corresponding to (**a**) at 10 ms intervals. (**c**) Transient behaviour for both emitter temperature and radiated power (using piecewise power series) for a 10101 serial input data sequence when modulated at 100 Hz, from OFF-state at ambient room temperature. (**d**) Time-variant power spectral density of thermal radiation corresponding to (**c**) at 10 ms intervals.

In practice, time is divided into short segments of Δ*t* (e.g., Δ*t* = 5 ms in this case) and this power series is reinitialized at the start of each time segment with a revised value of *T*(0) = *T*(Δ*t*), giving the piecewise power series result shown in Fig. 3a. Substituting Eq. (5) into Eq. (2) gives a time-dependent thermal emitter power spectral density, shown in Fig. 3b. The OOK modulation is essentially switching *W*_*e*_(*t*) between zero (OFF state for binary zero) and *W*_*R*_ (ON state for binary one), according to the serial input data stream. As an example, the transient behaviour of the thermal emitter for a ‘10101’ serial input data sequence, when modulated at 100 Hz, is given in Fig. 3c,d.

### Free space transmission

When propagating through free space, the thermally generated radiated power suffers from spreading loss and atmospheric attenuation. While spreading loss can be avoided by collimating the diverging thermal radiation beam, atmospheric attenuation is inevitable and frequency dependent. Figure 2a shows the simulated atmospheric transmittance ${{\mathscr{T}}}_{a}$ of a homogeneous free-space channel, under pristine atmospheric conditions, across a channel range *R*_*c*_ = 2 m. Mean channel transmittance ${{\mathscr{T}}}_{c}$, given by Eq. (6), is used to quantify a channel’s resilience to atmospheric attenuation:

6 $${{\mathscr{T}}}_{c}(T,{R}_{c})=\frac{{\int }_{0}^{+\infty }{p}_{e}(\lambda ,T){{\mathscr{T}}}_{f}(\lambda ){{\mathscr{T}}}_{a}(\lambda ,{R}_{c})d\lambda }{{\int }_{0}^{+\infty }{p}_{e}(\lambda ,T){{\mathscr{T}}}_{f}(\lambda )d\lambda }$$

where, ${{\mathscr{T}}}_{f}(\lambda )$ is the transmittance of the channel’s BPF, shown in Fig. 2a. As showed in Fig. 2b, most channels have a relatively constant mean channel transmittance with range, by trying to avoid absorption lines. The result demonstrates that the ‘THz Torch’ system is resilient to atmospheric attenuation across short-range links. Channel 4 has been highlighted, as it has a noticeably lower mean channel transmittance due to atmospheric attenuation from water vapour absorption lines.

**Figure 4:**
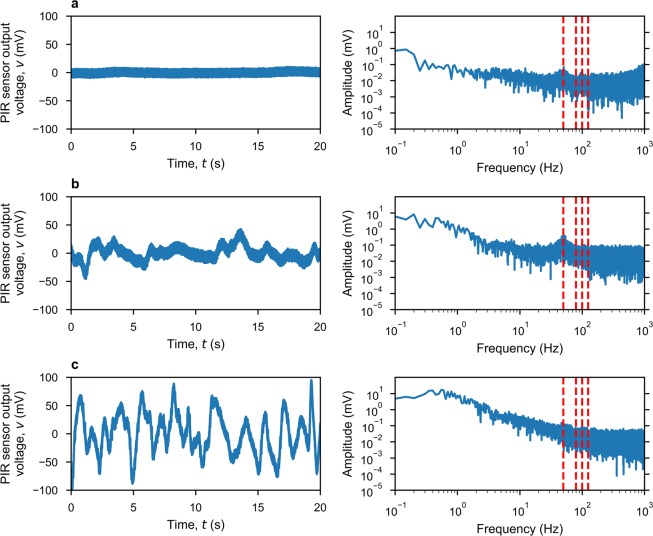
Measured thermal background noise and associated single-sided amplitude spectral responses (dashed red lines indicate different modulation frequencies) in different environments. (**a**) Inside an electromagnetically-shielded microwave anechoic chamber. (**b**) Large air-conditioned room. (**c**) Outdoors.

In practice, unwanted external noise/interference is also injected into the receiver, with contributions from: atmospheric noise, which is negligible because the ambient temperature and emissivity are much less than those of the thermal emitter; thermal convection on the surface of the PIR sensor; and radio frequency interference. A comprehensive study of all possible noise/interference sources and their influence on BER is beyond the scope of this paper. However, Figure 4a shows measured noise in three completely different environments: screen anechoic chamber, as a low noise reference; large air-conditioned office, as a typical indoor environment - used to obtain our experimental results; and an outdoor environment, in low wind conditions. The noise sources stated previously are clearly distinguishable from the amplitude spectral density shown in Fig. 4b. Thermal convection on the surface of the PIR sensor generates an undesirable temperature fluctuation, which contributes to low frequency noise up to *ca*. 10 Hz. This noise is most significant in an outdoor environment. Further work is needed to minimize this external noise source by shielding the sensor. Internal electronic noise, which comes from within the sensor itself and its backend electronics, can be seen above *ca*. 100 Hz; this has been previously investigated^14^. From *ca*. 10 to 100 Hz, a noise peak centered at 50 Hz can be observed from both indoor environments. This is attributed to 50 Hz ‘mains supply’ picked-up, as external radio frequency interference that is inter-modulated with internal electronic noise.

### Pyroelectric sensor

**Figure 5:**
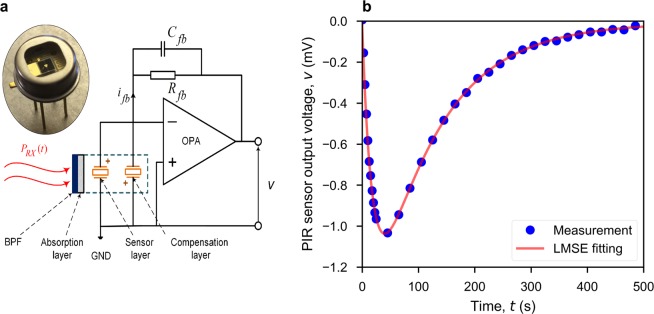
Pyroelectric infrared sensor (LME-335). (**a**) Equivalent circuit model. (**b**) Measured and fitted sensor output voltage, using Eq. (13), for a thermal radiation step response with a steady-state emitted temperature of 1,023 K for a single-channel 2 m link.

PIR sensors^19–21^ are commonly used in low-end applications, such as ubiquitous motion detection. However, the thermodynamic model developed in this section opens-up the PIR sensor to new applications; for example, in wireless receivers as an infrared radiation power-to-voltage transducer. The current-mode PIR sensor and its equivalent circuit model are shown in Fig. 5a. A channel BPF first rejects out-of-band power, allowing only the in-band radiation to be incident onto the sensor’s absorption layer. The resulting temporal temperature gradient generates pyroelectic current, by altering the electrical polarization magnitude within the pyroelectric layer. A compensation layer that is not exposed to the incident radiation is connected in parallel with the sensor, having an inverse polarity, to cancel-out environmental temperature drifts. Current flows through the feedback network of the operational amplifier (OPA), to give an output voltage. This combination of thermodynamics and transient behaviour of the circuit is represented by Eq. (7).

7 $$\begin{array}{ll}{\eta }_{p}{P}_{RX}(t)\,= & {H}_{p}\frac{d{T}_{p}(t)}{dt}+{G}_{p}{T}_{p}(t)\ (a)\\ q(t)\,= & p{A}_{p}{T}_{p}(t)\,(b)\\ {i}_{fb}(t)\,= & \frac{dq(t)}{dt}\,(c)\\ -{i}_{fb}(t)\,= & {C}_{fb}\cdot \frac{dv(t)}{dt}+\frac{v(t)}{{R}_{fb}}\,(d)\end{array}$$

where *η*_*p*_ is the absorptance of the absorption layer; *P*_*RX*_ is the incident radiated power onto the sensor; *H*_*p*_, *G*_*p*_ and *A*_*p*_ are the heat capacity, thermal conductance and surface area, respectively, of the pyroelectric layer; *T*_*p*_ is the temperature difference between the sensor and its metal encapsulation, which is assumed to be a heatsink at constant ambient temperature; *p* and *q* are the pyroelectric coefficient and generated charge, respectively, for the pyroelectric material; *i*_*fb*_, *R*_*fb*_ and *C*_*fb*_ are the feedback current, resistor and capacitor, respectively; *v* is the output voltage of the sensor. Equation (7a) follows the principle of conservation of energy, where the absorbed incident power (left-hand side) is represented by contributions from the sensor’s transient temperature and its rate of change. The pyroelectric effect is expressed by Eq. (7b). By re-arranging Eq. (7), the output voltage can be solved from:

8 $$\begin{array}{ccc}-{\eta }_{p}{P}_{RX}^{{\rm{{\prime} }}}(t) & = & \frac{{H}_{p}{C}_{fb}}{p{A}_{p}}{v}^{{\rm{{\prime} }}{\rm{{\prime} }}}(t)+\left[\frac{{H}_{p}}{p{A}_{p}{R}_{fb}},+,\frac{{G}_{p}{C}_{fb}}{p{A}_{p}}\right]{v}^{{\rm{{\prime} }}}(t)+\frac{{G}_{p}}{p{A}_{p}{R}_{fb}}v(t)\\ {P}_{RX}^{{\rm{{\prime} }}}(t) & = & \frac{d{P}_{RX}(t)}{dt}\,{v}^{{\rm{{\prime} }}{\rm{{\prime} }}}(t)=\frac{{d}^{2}v(t)}{d{t}^{2}}\,{v}^{{\rm{{\prime} }}}(t)=\frac{dv(t)}{dt}\end{array}$$

Transforming both sides of Eq. (8) into the Laplace-domain gives:

9 $$-{\eta }_{p}[s{P}_{RX}(s)-{P}_{RX}(0)]=\frac{{H}_{p}{C}_{fb}}{p{A}_{p}}[{s}^{2}v(s)-sv(0)-{v}^{{\rm{{\prime} }}}(0)]+\left[\frac{{H}_{p}}{p{A}_{p}{R}_{fb}},+,\frac{{G}_{p}{C}_{fb}}{p{A}_{p}}\right][sv(s)-v(0)]+\frac{{G}_{p}}{p{A}_{p}{R}_{fb}}v(s)$$

where *v*(0), ${v}^{{\rm{{\prime} }}}(0)$ and *P*_*RX*_(0) are initial conditions within the time-domain. The output voltage *v*(*s*) in the Laplace-domain can then be solved and transformed back into the time-domain *v*(*t*). However, the incident power *P*_*RX*_ does not normally have a closed-form expression in either the time- or Laplace-domains. As a result, a numerical solution, or power series approximation, similar to Eq. (5), can be adopted. The direct measurement or estimation of all parameters found in Eq. (7) can be problematic. To this end, a novel calibration approach is proposed, based on a step-response measurement, which enables the use of our derived thermodynamics to implement a form of ‘cognitive demodulation’. In the time-domain, the step incident power, having an amplitude power *P*_*step*_, is defined by:

10 $${P}_{RX}(t)=\left\{\begin{array}{ll}0 & {\rm{when}}\ t\le 0\\ {P}_{step} & {\rm{when}}\ t > 0\end{array}\right.$$

Substituting the Laplace transform of the step incident power ${P}_{RX}(s)=\frac{{P}_{step}}{s}$, and initial incident power *P*_*RX*_(0) = 0 into Eq. (9), the step response in the Laplace-domain *v*_*step*_(*s*) is:

11 $$\begin{array}{c}{v}_{step}(s)=-\frac{{a}_{1}v(0)s+{a}_{1}{v}^{{\rm{{\prime} }}}(0)+{a}_{2}v(0)+{\eta }_{p}{P}_{step}}{{a}_{1}{s}^{2}+{a}_{2}s+{a}_{3}}\\ {a}_{1}=\frac{{H}_{p}{C}_{fb}}{p{A}_{p}}\,{a}_{2}=\frac{{H}_{p}}{p{A}_{p}{R}_{fb}}+\frac{{G}_{p}{C}_{fb}}{p{A}_{p}}\,{a}_{3}=\frac{{G}_{p}}{p{A}_{p}{R}_{fb}}\end{array}$$

Transforming the solution back into the time-domain gives:

12 $$\begin{array}{ccc}{v}_{step}(t) & = & \frac{[{G}_{p}{R}_{fb}{C}_{fb}v(0)+{H}_{p}{R}_{fb}{C}_{fb}{v}^{{\rm{{\prime} }}}(0)+p{A}_{p}{R}_{fb}{\eta }_{p}{P}_{step}]{e}^{-\left(\frac{1}{{R}_{fb}{C}_{fb}}\right)t}}{{H}_{p}-{G}_{p}{R}_{fb}{C}_{fb}}\\  & - & \frac{[{H}_{p}v(0)+{H}_{p}{R}_{fb}{C}_{fb}{v}^{{\rm{{\prime} }}}(0)+p{A}_{p}{R}_{fb}{\eta }_{p}{P}_{step}]{e}^{-\left(\frac{{G}_{p}}{{H}_{p}}\right)t}}{{H}_{p}-{G}_{p}{R}_{fb}{C}_{fb}}\end{array}$$

With initial response $v(0)={v}^{{\rm{{\prime} }}}(0)=0$, Eq. (12) can be simplified to:

13 $$\begin{array}{c}{v}_{step}(t)={p}_{0}\cdot ({e}^{-{p}_{1}t}-{e}^{-{p}_{2}t})\\ {p}_{0}=\frac{p{A}_{p}{R}_{fb}{\eta }_{p}{P}_{step}}{{H}_{p}-{G}_{p}{R}_{fb}{C}_{fb}}\,{p}_{1}=\frac{1}{{R}_{fb}{C}_{fb}}\,{p}_{2}=\frac{{G}_{p}}{{H}_{p}}\end{array}$$

The step-response includes two exponential terms, corresponding to rising and recovery phases. To estimate the values of {*p*_0_, *p*_1_, *p*_2_}, one can measure the step-response directly and then fit to Eq. (13). This curve fitting represents a nonlinear least-mean-square-error (LMSE) problem, which can be solved by the Levenberg-Marquard algorithm. Figure 5b shows excellent agreement between the measured and fitted curves, demonstrating the effectiveness of our thermodynamic pyroelectric model. As illustrated in Fig. 1, the hardware setup for a step-response measurement is exactly the same as that for data transfer. Therefore, calibration of the sensor can be performed as a regular routine, which makes the communications link robust to channel environmental changes and component ageing. With parameters {*p*_0_, *p*_1_, *p*_2_}, Equation (8) can be reformulated as:

14 $$-{v}^{{\rm{{\prime} }}{\rm{{\prime} }}}(t)=({p}_{1}+{p}_{2}){v}^{{\rm{{\prime} }}}(t)+{p}_{1}{p}_{2}v(t)+{p}_{0}{\hat{P}}_{RX}^{{\rm{{\prime} }}}({p}_{1}-{p}_{2})$$

where ${\widehat{P}}_{RX}=\frac{{P}_{RX}}{{P}_{step}}$ is the normalized incident power and ${\widehat{P}}_{RX}^{{\prime} }$ is its gradient with respect to time. Calibrated values of {*p*_0_, *p*_1_, *p*_2_} now fully define the thermodynamic behavior of the pyroelectric sensor. *P*_*step*_ can be estimated by:

15 $${P}_{step}={L}_{c}{\int }_{0}^{+\infty }{p}_{e}(\lambda ,{T}_{s}){{\mathscr{T}}}_{f}^{2}(\lambda ){{\mathscr{T}}}_{l}^{2}(\lambda ){{\mathscr{T}}}_{a}(\lambda ,{R}_{c})d\lambda $$

From Eq. (2), *p*_*e*_(*λ*, *T*_*s*_) is the thermal emitter power spectral density at steady-state temperature *T*_*s*_; ${{\mathscr{T}}}_{l}$ is the transmittance of the lens. *L*_*c*_ represents all the uncharacterized frequency-independent losses (e.g., conical horn losses, filter aperture blockage, optical misalignment; and spreading loss if a lens is not used). When the thermal emitter operates at temperature *T*, the normalized incident power onto the sensor ${\widehat{P}}_{RX}$ is given by:

16 $${\widehat{P}}_{RX}(T)=\frac{{L}_{c}{\int }_{0}^{+\infty }{p}_{e}(\lambda ,T){{\mathscr{T}}}_{f}^{2}(\lambda ){{\mathscr{T}}}_{l}^{2}(\lambda ){{\mathscr{T}}}_{a}(\lambda ,{R}_{c})d\lambda }{{P}_{step}}=\frac{{\int }_{0}^{+\infty }{p}_{e}(\lambda ,T){{\mathscr{T}}}_{f}^{2}(\lambda ){{\mathscr{T}}}_{l}^{2}(\lambda ){{\mathscr{T}}}_{a}(\lambda ,{R}_{c})d\lambda }{{\int }_{0}^{+\infty }{p}_{e}(\lambda ,{T}_{s}){{\mathscr{T}}}_{f}^{2}(\lambda ){{\mathscr{T}}}_{l}^{2}(\lambda ){{\mathscr{T}}}_{a}(\lambda ,{R}_{c})d\lambda }$$

In Equation (16), the hard to characterize *L*_*c*_ term is conveniently cancelled. Moreover, a trapezoidal approximation can be used to numerically compute the integration in the numerator of Eq. (16).

### Digital receiver and cognitive demodulation

An optimal receiver generally employs matched filters, which can be interpreted as ‘template matching’ to noiseless copies of the received signal that correspond to all possible transmit waveforms. Demodulation can then be performed using the maximum likelihood decision rule. With the conventional approach, the templates are time-invariant functions and the matched filter employs cross-correlation^18^. In contrast, our system employs a linear combination of Pearson correlation and Euclidean distance for time-variant template matching. Once again, our uniquely thermal-based transmit-receiver physical layer naturally lends itself to this solution.

Cognition encompasses many aspects of intellectual functions and processes, such as reasoning, computation and decision making. Cognitive processes use existing knowledge to generate new knowledge. In engineering, one example is cognitive radio^22^; whereby a radio detects available channels within the wireless spectrum and then changes its transmission or reception parameters accordingly to perform dynamic spectrum management. In our case, cognitive demodulation dynamically makes a decision on the start of frame and predicts both binary states for each bit within the frame. Our concept of ‘Cognitive Demodulation’ is justified for the following reasons. First, our solution constantly tracks the thermodynamics of the source and detector, to generate time-variant templates. Second, within the time-variant template predictions, an additional step-response calibration characterizes the transmit-receive channel to update the thermodynamic model parameters.

The output voltage of the pyroelectric sensor is sampled by an ADC and processed by the digital receiver. For the ‘THz Torch’ wireless link to allow asynchronous operation, the receiver first needs to detect the start of a frame, for synchronization, so that it can demodulate the data within these frames. Two methods, operating in the frequency- and time-domains, are combined to guarantee stable synchronization. First, the frequency-domain method is based on a discrete short-term Fourier transform (STFT), represented by Eq. (17).

17 $$STFT\{v[t]\}(\tau ,\omega )=V[\tau ,\omega ]=\mathop{\sum }\limits_{t=-\infty }^{+\infty }v[t]w[t-\tau ]{e}^{-j\omega t}$$

Here, the signal is truncated with a sliding rectangular window function *w*[*t* − *τ*], shifted in time by *τ*, and then the conventional discrete Fourier transform (DFT) is performed. The rationale behind this method is that the output voltage from the sensor can be considered as a non-stationary signal having its discrete frequency components that vary in time. An idle link generates an output voltage that is mainly composed of a direct-current (DC) offset voltage and noise (as seen in Fig. 4). When a frame starts, the spectral power density from the STFT increases significantly at the modulation frequency.

A time-domain approach for frame synchronization is also implemented, inspired by our cognitive demodulation algorithm (discussed more later). The waveform corresponding to the header of a frame is predicted in advance. The measured output signal from the sensor is truncated by another sliding rectangular window function, which has the same duration as the predicted waveform of the header. When the truncated signal matches the predicted waveform, the current location of the sliding window indicates the start of a frame and, thus, synchronization is established.

Measurement-prediction matching is achieved by calculating Eq. (18), which represents the dissimilarity between two time series, *x* and *y*, having the same length. It is essentially a linear combination of Pearson correlation (first term), with weighting parameter *w*, and Euclidean distance (second term).

18 $$S(x,y)=w\frac{{\sum }_{i=1}^{n}({x}_{i}-\bar{x})({y}_{i}-\bar{y})}{\sqrt{{\sum }_{i=1}^{n}{({x}_{i}-\bar{x})}^{2}}\sqrt{\mathop{\sum }\limits_{i=1}^{n}{({y}_{i}-\bar{y})}^{2}}}+\mathop{\sum }\limits_{i=1}^{n}{({y}_{i}-{x}_{i})}^{2}$$

Once frame synchronization has been established, the receiver will record the sensor output until the end of the current frame and divide-up the whole sequence into equal segments, each corresponding to a bit. For each bit, the receiver employs techniques described previously to make two predictions. The thermal emitter temperature *T*(*t*) during the transmission of a bit can be predicted using Eq. (5). With the OOK modulation scheme, *W*_*e*_ in Eq. (5) is forced to equal either the rated power *W*_*R*_ or zero, to predict a binary one or binary zero, respectively. The normalized incident power received by the pyroelectric sensor ${\widehat{P}}_{RX}(t)$ can be predicted by substituting *T* by *T*(*t*) in Eq. (16). With ${\widehat{P}}_{RX}(t)$, Equation (14) can be numerically solved, which gives two predictions corresponding to binary one and zero. Input serial data is then demodulated by matching the measured segment to one of the predictions.

**Figure 6:**
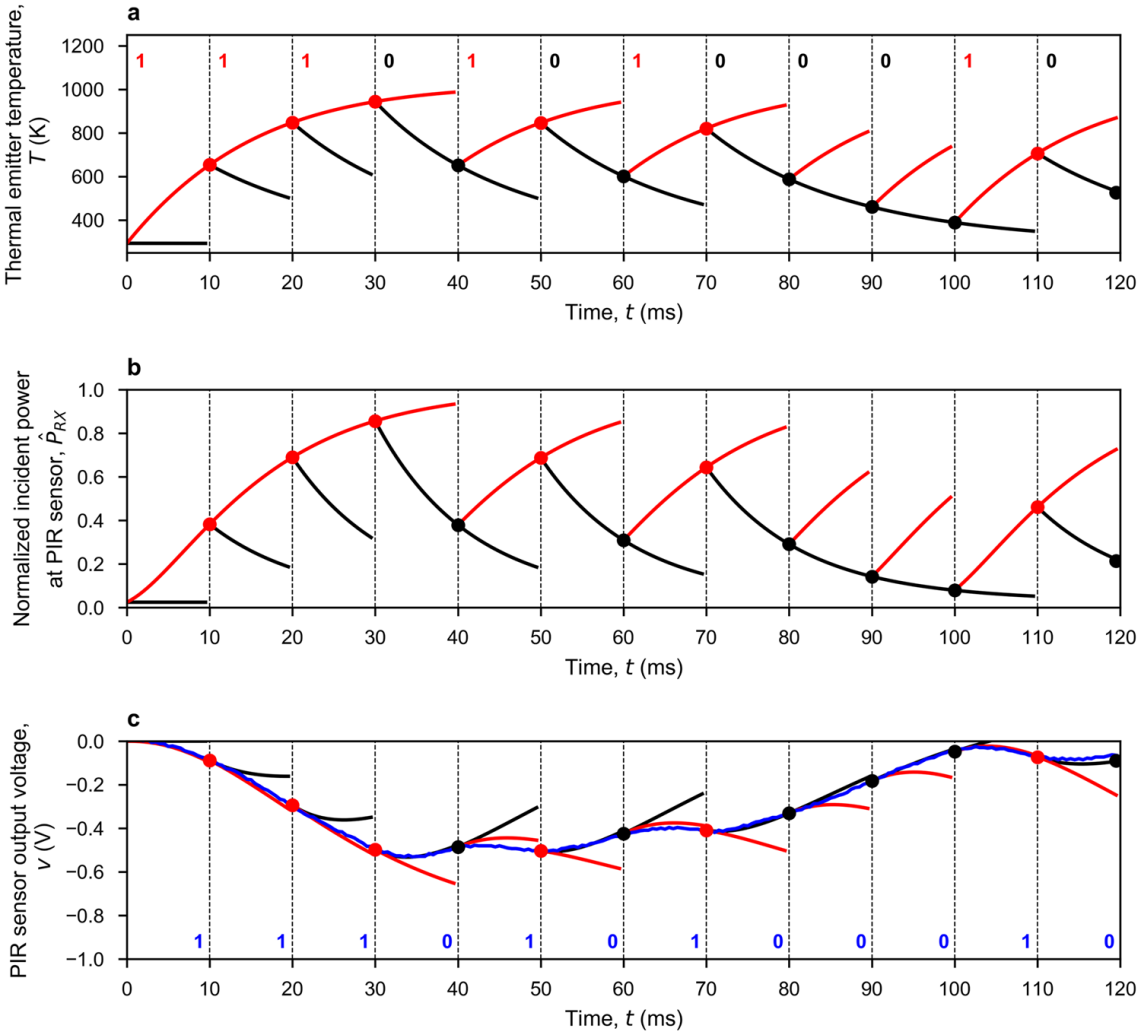
Operation of the cognitive demodulation algorithm for a single-channel 2 m link, with arbitrarily chosen ‘111010100010’ serial input data sequence sent at 100 bps, initially from OFF-state at ambient room temperature, with both ON-state (red) and OFF-state (black) predictions. (**a**) Calculated sequence of thermal transients, using Eq. (5), at the thermal emitter. (**b**) Calculated normalized incident power, using Eq. (16), at the PIR sensor. (**c**) PIR sensor output voltage predictions (red and black) and measurements (blue), with resulting demodulated serial output data sequence showing no errors.

**Figure 7:**
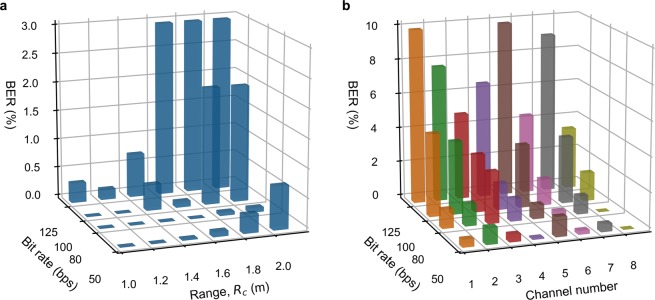
Measured raw bit error rate performance: (**a**) Single-channel BER performance across 1 to 2 m links. (**b**) Multi-channel BER performance for each channels across a 0.5 m link.

Solving these thermodynamic equations, for making predictions, requires initial conditions. For the first bit within a frame, the initial temperature *T*(0) for Eq. (5) is set at the ambient temperature *T*_0_; while *v*(0) and ${v}^{{\prime} }(0)$ in Eq. (14) are set to zero. For the following bits, the initial conditions can be obtained from the matched prediction of its previous bit. This procedure is run iteratively until the whole frame is demodulated. Figure 6 shows an example of our cognitive demodulation algorithm, based on real measured data. Figure 7 shows the measured BER, using cognitive demodulation, for both single-channel and multi-channel prototype links. As expected, BER increases with bit rate and range. However, with the former, there is also a degradation at 50 bps due to external 50 Hz interference from mains power supply pick-up.

## Discussion

In this work, a new method of demodulation is presented for thermal-based ‘THz Torch’ digital communications links, based on device thermodynamics. By modelling the transient behavior of the thermal emitter and pyroelectric sensor, our cognitive demodulation has resulted in a major step-change improvement in the operational figure of merit (Range  ×  Bit Rate), as demonstrated by proof-of-principle single-channel and multi-channel asynchronous ‘THz Torch’ wireless links.

An implementation and performance summary comparison of the reported ‘THz Torch’ wireless links is given in Supplementary Table S2. It can be seen that the work presented in this paper not only allows for truly asynchronous operation, but when sending arbitrary data, gives a five-fold increase in the operational figure of merit when compared to using indirect modulation (mechanical chopper) and 10,000-fold increase when compared to using direct modulation (electronic modulation).

By scaling-up the effective sizes of the emitter and pyroelectric sensor areas, all performance characteristics can be improved; offering real-world applications for secure communications. It is believed that this work can be extended to bolometers (and even microbolometer arrays employed in terahertz/infrared cameras), having applications that currently rely on threshold detection, but with response times that are limited by their thermal time constants. Moreover, while our application has been applied to binary modulation, more predictions could be formulated for M-ary amplitude shift keying signaling.

## Methods

### The ‘THz Torch’ prototype setup

The transmitter is composed of a commercial infrared thermal emitter (INTX 17-0900) modulated by a switchable constant current source (RCD-24-0.70), to deliver the typical quoted value of 117 mA, required for the operating steady-state temperature of 1,023 K. To modulate the thermal emitter according to the serial digital bit steam, a microprogrammed control unit (Arduino-DUE) is employed to switch the driver (current source) between the ON/OFF states. For the single channel prototype, the noise carrier is defined by a matched pair of 8–14 *μ*m silicon wide-BPFs. The multi-channel prototype exploits eight matched pairs of coated narrow-BPFs, fabricated by Northumbria Optical Coatings Ltd. A matched pair of ZnSe lenses are used for beam collimation (Tx) and focusing (Rx). At the receiver, incident radiated power is converted to a voltage by a pyroelectric sensor (LME-335). The signal is then sampled by a data acquisition card (NI USB-6009) and sent to a computer to perform cognitive demodulation.

### Thermal infrared emitter

The first proof-of-principle demonstrator^11^, using direct electronic modulation is limited by the thermal time constants of the emitter (e.g., for a miniature incandescent light bulb, the previously reported turn-ON rise time (10% to 90%) was 645 ms; while the turn-OFF decay time (90% to 10%) was 2,415 ms). By comparison, the commercial thermal infrared emitter used here (INTX 17-0900) has turn-ON and turn-OFF thermal time constants (defined by the time needed to reach 63.2% of the steady state) of only 14.4 ms and 16.8 ms, respectively.

In steady state, the INTX 17-0900 emitter requires a typical value of *W*_*R*_ = 897.8 mW to keep its temperature at *T*_*s*_ = 1,023 K. With its emissivity *ε* = 0.8 and heated membrane area *A*_*e*_ = 2.89 mm^2^, equation (4) can be used to estimate its thermal conductivity *k*_*e*_ = 1.03e^−3^ W/K. Given its measured thermal time constant of 14.4 ms, a grid search method is used to obtain the heat capacity *C*_*e*_ = 1.9e^−5^ J/K.

### Atmospheric attenuation simulation

The atmospheric attenuation originates from the absorption and scattering caused by molecules, particles and liquid droplets. Previous work^23^ has demonstrated the effectiveness of modelling atmospheric attenuation under pristine conditions (e.g., indoors and in dry environments outdoors) employing the ‘HITRAN on the Web’ simulator, using the U.S. Standard 1976 mid-latitude atmosphere model developed by NASA.

### Noise measurement

The noise measurement is undertaken by recording the output voltage from the sensor, over a period of 20 seconds, with a sampling frequency of 20 kHz.

### Pyroelectric sensor calibration

To generate the step incident power, used for sensor calibration, the optical shutter is first closed. The emitter is heated-up to its steady-state temperature and then the shutter is opened for 600 ms. Instead of fitting the measured step response directly to Eq. (13), in practice, an additional term *p*_3_ is added to compensate for any offset voltage in the output of the OPA. This curve fitting can be transformed into a nonlinear least-square problem, which can be solved using the Levenberg-Marquardt algorithm. Without suitable initial values, convergence to the global optimum cannot be guarantee. For example, with our PIR sensor, initial values *p*_1_ = 50 and *p*_2_ = 5 can be estimated using Eq. (13) with the following typical parameter values: *R*_*fb*_ = 100 GΩ, *C*_*fb*_ = 0.2 pF, *G*_*p*_ = 4.32 mW/K and *H*_*p*_ = 0.864 mJ/K, given by the sensor manufacturer. These initial values can be compared to the following fitted values from the calibration measurement, with Eq. (13) and the additional *p*_3_ term: *p*_0_ ~  1.59 V, *p*_1_ ~  49.48 s^-1^, *p*_2_ ~  10.26 s^-1^ and *p*_3_ ~ −0.01 V.

### BER measurement

The minimum transmission unit is represented by a frame. In practical applications, the frame could mimic the simplex universal asynchronous receiver-transmitter (UART) format; having a start bit, eight data bits (least significant bit first), a parity bit and stop bit(s). However, here, the raw (i.e, without applying error correction) bit error rate is measured by sending 2,000 frames, each of which contains a four start bit header ‘1110’ and 50 random data bits, giving a total of 0.1 million data bits.

## Supplementary information

Supplementary Tables

Supplementary Video
