# Supplementary material for: Thermodynamics-based Cognitive Demodulation for 'THz Torch' Wireless Communications Links
Source: Sci Rep. 2020 Apr 10;10:6259. doi: 10.1038/s41598-020-62234-1 (PMC7148299; doi:10.1038/s41598-020-62234-1)
Supplement: Supplementary file 1 — Supplementary Tables [file 41598_2020_62234_MOESM1_ESM.pdf]

Thermodynamics-based Cognitive Demodulation for ‘THz Torch’ Wireless Communications Links

Hang Ren[1] **and Stepan Lucyszyn**[1,*]

[1]Department of Electrical and Electronic Engineering, Imperial College London, London, SW7 2AZ, UK
[*]s.lucyszyn@imperial.ac.uk

Supplementary Table

Supplementary Table S1. Band-pass filter specifications

	50% cut-off, λ_1 to λ_2, bandwidth μm (THz)	Aperture size mm^2	Aperture blockage* %
ch#1	4.48 - 4.54 (65.95 - 66.88)	4.6×4.6	67
ch#2	4.74 - 4.79 (62.57 - 63.25)	4.9×4.9	63
ch#3	5.09 - 5.31 (56.44 - 58.83)	3.8×3.8	78
ch#4	7.01 - 7.11 (42.19 - 42.79)	5.6×5.6	51
ch#5	7.37 - 7.48 (40.08 - 40.67)	5.6×5.6	51
ch#6	8.22 - 8.62 (34.81 - 36.45)	3.8×3.8	78
ch#7	9.61 - 10.03 (29.92 - 31.23)	3.7×3.7	69
ch#8	10.09 - 10.54 (28.46 - 29.74)	4.7×4.7	65
Single	8.03 - 13.74 (21.83 - 37.33)	4.6×4.6	67

* With reference to an unblocked circular filter having diameter $\varnothing = 9$ mm

Supplementary Table S2. Implementation and performance summary comparisons of reported 'THz Torch' wireless links

	Sep. 2011[1]	Dec. 2011[2]	Apr. 2013[3]	Jun. 2014[4]	May 2016[5]	This Work
Source Type	5×Eiko-8666<br>$4.7 \times 3.7\ mm^2$	5×Eiko-8666<br>$4.7 \times 3.7\ mm^2$	5×Eiko-8666<br>$4.7 \times 3.7\ mm^2$	5×Eiko-8666<br>$4.7 \times 3.7\ mm^2$	5×Eiko-8666<br>$4.7 \times 3.7\ mm^2$	INTX 17-0900<br>$1.7 \times 1.7\ mm^2$
Emitter DC Power (mW)	146	4×146 = 584	146	4×994 = 3,976	994	898 and<br>8×898 = 7,184
Number of Channels	1	4	1	4	1	1 and 8
3 dB Bandwidth (THz)	25	∼4×15 = 60	25	∼4×15 = 60	25	16 and<br>∼8×2.5
Modulation Mechanism	Electronic	Chopper	Chopper	Chopper	Chopper	Electronic
Transmitted Data	Clock	Clock	Clock	Arbitrary	Clock	Arbitrary
Operation	Record and<br>post-process	Record and<br>post-process	Record and<br>post-process	Record and<br>post-process	Record and<br>post-process	Fully<br>asynchronous
Tx-Rx Lens Pair	None	None	None	None	KBr<br>∅1.5 cm	ZnSe<br>∅2.5 cm
min ⇒ max Range (cm)	0.5 ⇒ 2.5	1.0	1.0	3 ⇒ 4	12 ⇒ 15	100 ⇒ 200<br>and 50
PIR Sensor	Voltage mode<br>IRA-E710ST1	Voltage mode<br>IRA-E710ST1	Current mode<br>LME-553	Current mode<br>LME-553	Current mode<br>LME-553	Current mode<br>LME-335
Total NRZ Bit Rate (bps)	10.2 ⇒ 1	4×20.6 = 82	760	4×640 = 2,560	2,000	125 and<br>8×125 = 1,000
Figure of Merit<br>(cm × bps)	5.1	82	760	10,240	30,000	25,000<br>and 50,000
Bit Error Rate (%)	0<br>qualitative	0<br>qualitative	0<br>qualitative	0.11 ⇒ 15<br>quantitative	0.0018 ⇒ 18<br>quantitative	see Figure<br>quantitative

References

1. Lucyszyn, S., Lu, H. & Hu, F. Ultra-low cost thz short-range wireless link. In *IEEE MTT-S International Microwave Workshop Series on Millimeter Wave Integration Technologies*, 49–52 (IEEE, 2011).

2. Hu, F. & Lucyszyn, S. Ultra-low cost ubiquitous thz security systems. In *Asia-Pacific Microwave Conference 2011*, 60–62 (IEEE, 2011).

3. Hu, F. & Lucyszyn, S. Improved 'thz torch' technology for short-range wireless data transfer. In *2013 IEEE International Wireless Symposium*, 1–4 (IEEE, 2013).

4. Liang, X., Hu, F., Yan, Y. & Lucyszyn, S. Secure thermal infrared communications using engineered blackbody radiation. *Sci. Reports* **4**, 5245 (2014).

5. Hu, F. & Lucyszyn, S. Advances in front-end enabling technologies for thermal infrared 'thz torch' wireless communications. *J. Infrared, Millimeter, Terahertz Waves* **37**, 881–893 (2016).
